# Septic shock: ECMO beyond ARDS? Introducing the Simon two-stage protocol when randomisation is considered unethical

**DOI:** 10.1186/s13049-020-0714-3

**Published:** 2020-03-17

**Authors:** Alexander Buia, Hans-Bernd Hopf, Eva Herrmann, Thomas Schmandra, Ernst Hanisch

**Affiliations:** 1 Department of General, Visceral and Thoracic Surgery, Asklepios Klinik Langen, Academic Teaching Hospital Goethe-University Frankfurt, Röntgenstr 20, 63225 Langen, Germany; 2 Department of Anaesthesia and Perioperative Medicine, Asklepios Klinik Langen, Academic Teaching Hospital Goethe-University Frankfurt, Röntgenstr 20, 63225 Langen, Germany; 3 Department of Biostatistics and Mathematical Modeling, Goethe-University Frankfurt, Theodor-Stern-Kai 7, 60590 Frankfurt, Germany; 4 Department of Vascular Surgery, Rhön Klinik Campus Bad Neustadt, Von-Guttenberg-Str. 11, 97616 Bad Neustadt a. d. Saale, Germany

One of the great disappointments during the past decades has been the failure to convert advances in our understanding of the biologic features of sepsis into new therapies. Although numerous multicenter, randomized controlled trials have been conducted in the last 30 years, no reduction of septic shock mortality was achieved [1] [2]. [3] Taking together available evidence suggests that no sepsis specific therapies are available until today, although a roadmap on the way to a specific septic shock therapy might be on the horizon [3] [4] including artificial intelligence [5]. [6].

Interestingly, in the Surviving Sepsis Campaign guidelines [7] ECMO (**E**xtra **C**orporal **M**embrane **O**xygenation) is not mentioned as a treatment option.

ECMO has been a standard treatment for cardiac and respiraory failure in neonates and children for decades, [8] in contrast to adults where the debate about the benefit of ECMO still is ongoing.

A systematic review and meta-analysis showed that the use of veno-arterial life support (ECLS) in cardiac arrest was associated with a higher survival (NNT [number needed to treat] 7.7) as well as a better neurological outcome, in the setting of cardiogenic shock there was a higher survival with ECLS compared with IABP (NNT 3) [9].

Since even the efficacy of venovenous extracorporal membrane oxygenation with severe respiratory distress syndrome (ARDS) has been disputed controversially until today a randomized controlled trial comparing vv-ECMO with conventional treatment was initiated with the results published recently (EOLIA trial) [10]. The main causes of ARDS were bacterial pneumonia in 45% and viral pneumonia in 18% of the patients, 78% of the patients had severe sepsis or septic shock. Primary end point was mortality at 60 days. No difference was found in the initial intention-to-treat analysis. However, 35 patients (28%) in the control group crossed over to ECMO. In fact, reanalysis including crossover patients as treatment failure and comparing death/crossover to ECMO of the control group vs death in the ECMO group showed a 23% lower mortality in the ECMO group (NNT 5).

In addition, Robert Bartlett in an editorial in CCM argued that the difference of the intention to treat vs treatment failure analysis results from 35 patients in the conventional care group who crossed over to the ECMO group as rescue treatment when conventional care was failing. Accordingly, the study had become a study of early versus late ECMO. Therefore, the EOLIA trial had shown that ECMO should be used promptly when high-risk criteria are met, rather than as late rescue therapy when death from ARDS or multiple organ failure is imminent [11].

What about the use of va-ECMO in the treatment of septic shock? To date, ECMO is suggested only as rescue therapy [12] and only observational case series with some promising results have been published (for overview see Table 1). However, the impression is not unequivocal. Thus, in a commentary discussing the results of Ro et al. the question is asked whether ECMO therapy in septic shock is a heroic futility or not [27]. The authors come to the conclusion that in this study more than half of the patients had liver cirrhosis, including post-liver transplantation and the patient cohort was derived from the years 2005 through 2012; both factors could adversely affect outcomes. On the contrary, the results of a just recently published observational study [26] are very promising with a hospitality mortality of just 10% in cytotoxic cardiac failure and 30% in distributive shock.

**Table 1:** Observational studies using ECMO in adult septic shock, modified and extended according to Riera [13] et al.

Study	N	Sites of infection %	ECMO configuration	Hospital Survival %
Brechot et al. [14]	14	Pneumonia bacterial 79 Abdomen 14,3	Peripheral va 36% Switch to vv	71
Huang et al. [15]	52	Pneumonia 48 Abdomen 23 Urinary tract 9,6	Peripheral va	15
Cheng et al. [16]	108	Pneumonia bacterial 40 Myocarditis 25 Primary bloodstream 19,4	Peripheral va 78,7 Peripheral vv 21,3	28,7
Park et al. [17]	32	Pneumonia bacterial 34,4 Abdomen 21,9 Urinary tract 12,5	Peripheral va	21,9
Cheng et al. [18]	151	Pneumonia 50,3 Myocarditis 19,9 Bloodstream 14,6 Abdomen 7,6	Peripheral va 66,9 vv 33,1	29,8
Von Bahr et al. [19]	255	Pneumonia bacterial 53 Pneumonia viral 12,5	vv 68 switch to va peripheral 21 switch to vv 16	64
Takauji et al. [20]	40	Pneumonia 62,5 Abdomen 20 Urinary tract 7,5	vv	47,5
Yeo et al. [21]	8	Pneumonia *n* = 5 Extra-pulmonary *n* = 3	vva	50
Choi et al. [22]	28	Pulmonary 46,4 Abdomen 14,3 Genitourinary 7,1 Skin or soft tissue 14,3 Cardiovascular 7,1 Central nerve system 3,6 Catheter-induced 3,6 Other 3,6	vv, va, vva	35,7
Ro et al. [23]	71	Pneumonia 70 Abdomen 11 Urinary tract 5 Other 14	va	7
Vogel et al. [24]	12	Septic cardiomyopathy *n* = 12	vva	75
Banjas et al. [25]	19	Pneumonia 53 Abdomen 42 Soft tissue 5	vv, va, vva	42
Falk et al. [26]	37	Lung *n* = 21 Gut *n* = 2 Pyelonephritis *n* = 4 Fasciitis *n* = 4 Urine n = 1 Blood n = 4 Myocarditis n = 1	vv, va	Distributive shock 70,6 Cytotoxic Cardiac failure 90,0

In this situation where randomized controlled trials are considered unethical, Bartlett suggests that the matched pairs method is the best study design for evaluation of life support systems in acute fatal illness in a high risk population early [8].

For patients with septic shock we suggest another approach – the Simon [28] two-stage non-randomized method including only patients with septic shock (Sepsis-3 definition [7];) resulting from secondary peritonitis.

The Simon protocol is a Phase 2 design. According to Evrard et al. “Phase 2 is a completely underutilized tool in surgery that could raise the level of scientific reporting. The threshold for efficacy and nonefficacy is defined and with statistical power (risks *a* and *b*), the cohort size is calculated (usually, between 30 and 60 patients). The use of interim stopping rules allows reduction of the required number of patients (e.g., Simon design)” [29].

We use the following assumptions: ECMO treatment is considered as poor (P_0_) if mortality is above 55% and as sufficient (P_1_) if it is below 40% with a two-sided alpha of 0.05 and a beta of 0.20.

Inclusion criteria: Overcoming the criticism of sepsis trials in the past, namely heterogeneity of patient populations (ie mixing medical and surgical patients), we are only studying septic shock due to secondary peritonitis, a group with a reported very high mortality; age: 18–70 years, norepinephrine > 0,5 μg/kg/min despite adequate volume substitution (30 ml/kg) within the first 12 h, serum lactate > 2 mmol/l, duration of septic shock < 24 h (ie ECMO is initiated as an *early* and not as a rescue therapy); Exclusion criteria: Septic shock due to other sources than the abdomen, peripheral arterial occlusive disease excluding femoral/axillary vessel cannulation; duration of septic shock > 24 h; patient waiver concerning life support measures.

The ECMO route is peripheral with an initial flow ≥4.5 l/min and norepinephrine should be reduced at least to below norepinephrine > 0,5 μg/kg/min. The standard protocol in patients with VA-ECMO is to adjust the blood flow of the machine and the dosage of norepinephrine in order to achieve a mean arterial pressure of at least 65 mmHg. After achieving this MAP norepinephrine is tapered if possible, to below 0.1 μg/kg/min. In addition, inferior vena cava saturation is monitored continuously with a target saturation above 55. Finally, transthoracic echocardiography is performed daily when norepinephrine dosage is above 0.5 μg/kg/min.

CRRT will be used while on ECMO (default CRRT is SLED, it might be possible to use the oxygenator for connecting the dialysis machine, depending on the arterial and internal pressure of the oxygenator, if too high, a separate dialysis catheter has to be established). Distal arterial limb perfusion is used in every patient by a 6F sheat strengthened by wire. Filters for removing cytokines are not allowed.

The design with a moderate cluster effect results in the following study conditions: Include 40 patients at a first stage and reject the approach if 16 or less patients survive (and 24 more die).

Otherwise, the second stage will be recruited until overall *n* = 79 patients are included. The treatment approach will be rejected if overall 43 or less patients survive (i. e., 36 or more patients die).

This study design follows important recommendations: First, it is not a randomized trial (ie avoiding ethical issues, [30] [8]; and second, the defined patient population has a high mortality risk and avoids heterogeneity. Moreover, the Simon approach leads rapidly to results in a small number of patients in which a minimum of data collection could be provided [31].

In conclusion in answering the question ‘Septic shock – ECMO beyond ARDS?’ we strongly believe, against the background of quite discrepant results of observational studies (see Table 1 [13])that va-ECMO should be studied as outlined in the above protocol with emphasis on *early* ECMO (ie not as *rescue* ECMO) when failure of conventional therapy for septic shock is evident: norepinephrine > 0,5 μg/kg/min despite adequate volume substitution (30 ml/kg) within the first 12 h and serum lactate > 2 mmol/l*.*

## Data Availability

Not applicable

## Abbreviations

ECMO: extra corporal membrane oxygenation
ARDS: Acute respiratory distress syndrome
va: veno-arterial
vv: venovenous
NNT: number needed to treat
RRT: renal replacement therapy
SLED: sustained low efficiency dialysis
